# (2*E*,5*E*)-2,5-Bis(3,4,5-trimethoxy­benzyl­idene)cyclo­penta­none

**DOI:** 10.1107/S1600536808029474

**Published:** 2008-09-20

**Authors:** Yi-Feng Sun, Yang Liu, Feng-Yu Zhang, Hong-Ji Chen, Yi-Ping Cui

**Affiliations:** a Advanced Photonics Center, School of Electronic Science and Engineering, Southeast University, 210096 Nanjing, Jiangsu, People’s Republic of China; b Department of Chemistry, Taishan University, 271021 Taian, Shandong, People’s Republic of China; c Library, Taishan University, 271021 Taian, Shandong, People’s Republic of China

## Abstract

The title compound, C_25_H_28_O_7_, was prepared by the base-catalysed reaction of 3,4,5-trimethoxy­benzaldehyde with cyclo­penta­none. The mol­ecule has crystallographic twofold rotation symmetry and adopts an *E*-configuration about the central olefinic bonds. The two benzene rings and the central cyclo­penta­none ring are almost coplanar [dihedral angle = 4.7 (2)°].

## Related literature

For background literature, see: Guilford *et al.* (1999 ▶); Xue *et al.* (2008 ▶); Wu *et al.* (2008 ▶); Das *et al.* (2008 ▶). For related crystal structures, see: Sun & Cui (2007 ▶); Du *et al.* (2007 ▶); Wei *et al.* (2008 ▶).
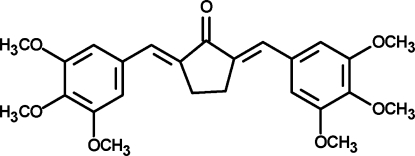


## Experimental

### 

#### Crystal data

C_25_H_28_O_7_
*M* *_r_* = 440.47
Monoclinic, 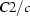

*a* = 18.573 (4) Å
*b* = 15.231 (3) Å
*c* = 8.8460 (18) Å
β = 113.99 (3)°
*V* = 2286.2 (10) Å^3^
*Z* = 4
Mo *K*α radiation
μ = 0.09 mm^−1^
*T* = 293 (2) K
0.30 × 0.20 × 0.20 mm

#### Data collection

Enraf–Nonius CAD-4 diffractometer
Absorption correction: ψ scan (North *et al.*, 1968 ▶) *T*_min_ = 0.973, *T*_max_ = 0.982
2123 measured reflections
2058 independent reflections
1422 reflections with *I* > 2σ(*I*)
*R*_int_ = 0.048
3 standard reflections every 200 reflections intensity decay: none

#### Refinement

*R*[*F* ^2^ > 2σ(*F* ^2^)] = 0.055
*wR*(*F* ^2^) = 0.164
*S* = 1.00
2058 reflections
146 parameters
H-atom parameters constrained
Δρ_max_ = 0.21 e Å^−3^
Δρ_min_ = −0.27 e Å^−3^

### 

Data collection: *CAD-4 Software* (Enraf–Nonius, 1989 ▶); cell refinement: *CAD-4 Software*; data reduction: *XCAD4* (Harms & Wocadlo, 1995 ▶); program(s) used to solve structure: *SHELXS97* (Sheldrick, 2008 ▶); program(s) used to refine structure: *SHELXL97* (Sheldrick, 2008 ▶); molecular graphics: *SHELXTL* (Sheldrick, 2008 ▶); software used to prepare material for publication: *SHELXTL*.

## Supplementary Material

Crystal structure: contains datablocks global, I. DOI: 10.1107/S1600536808029474/fj2151sup1.cif

Structure factors: contains datablocks I. DOI: 10.1107/S1600536808029474/fj2151Isup2.hkl

Additional supplementary materials:  crystallographic information; 3D view; checkCIF report

## Figures and Tables

**Table 1:** Selected torsion angles (°)

O1—C1—C2—C4	−2.7 (3)
C1—C2—C4—C5	178.0 (2)
C2—C4—C5—C6	179.6 (3)
C13—O4—C7—C6	1.8 (4)
C12—O3—C8—C7	86.9 (3)
C11—O2—C9—C8	174.8 (3)

**Fig. 1.**
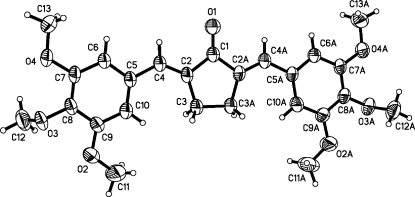
View of the molecule of (I) showing the atom-labelling scheme. Displacement ellipsoids are drawn at the 50% probability level. H atoms are shown as small spheres of arbitrary radii. Suffix A corresponds to symmetry code (-x+1, y, -z+1/2).

## supplementary crystallographic information

### Comment

Bis(arylmethylidene)cycloalkanones are widely used as building blocks for the synthesis of biologically active heterocycles (Guilford *et al.*, 1999), and reported to exhibit promising two-photon absorption (TPA) property (Xue *et al.*, 2008; Wu *et al.*, 2008). Moreover, it has been reported that some compounds containing the 3-(3,4,5-trimethoxyphenyl)-2-propenoyl group displayed potent multidrug resistance (MDR) reversal properties in cancer chemotherapy. In particular, 2,5-bis(3,4,5-trimethoxybenzylidene)cyclopentanone was 31 times more potent than verapamil as a MDR revertant (Das *et al.*, 2008). In this contribution, we report the crystal structure of the title compound, 2,5-bis(3,4,5-trimethoxybenzylidene) cyclopentanone, Fig.1.

The molecule possesses normal geometric parameters and adopts an E configuration about the central olefinic bonds (Fig. 1). The cyclopentanone ring and the two benzene rings are almost coplanar which allows conjugation. Among the six methoxy groups, only O3/C12 and O3A/C12A deviate from the molecule mean plane on the opposite side, the others are nearly coplanar with their attached benzene ring (Table 1).

Similar structures have been observed in the related substituted cyclohexanone and cyclopentanone analogues reported by Sun & Cui (2007), Du *et al.* (2007) and Wei *et al.* (2008).

### Experimental

The title compound was synthesized from cyclopenthexanone and 3,4,5-trimethoxybenzaldehyde as reported (Sun *et al.*, 2007).Yellow block crystals suitable for an X-ray structural analysis were obtained by slowly evaporating an ethanol solution at room temperature.

### Refinement

All H atoms were initially located in a difference Fourier map. The methyl H atoms were then constrained to an ideal geometry with C—H distances of 0.96 Å and *U*_iso_(H) = 1.5*U*_eq_(C). All other H atoms were placed in geometrically idealized positions and constrained to ride on their parent atoms with C—H distances 0.93–0.97 Å and *U*_iso_(H) = 1.2*U*_eq_(C).

### Crystal data

**Table:**

C_25_H_28_O_7_	*F*(000) = 936
*M**_r_* = 440.47	*D*_x_ = 1.280 Mg m^−^^3^
Monoclinic, *C*2/*c*	Mo *K*α radiation, λ = 0.71073 Å
Hall symbol: -C 2yc	Cell parameters from 25 reflections
*a* = 18.573 (4) Å	θ = 10–13°
*b* = 15.231 (3) Å	µ = 0.09 mm^−^^1^
*c* = 8.8460 (18) Å	*T* = 293 K
β = 113.99 (3)°	Block, yellow
*V* = 2286.2 (10) Å^3^	0.30 × 0.20 × 0.20 mm
*Z* = 4	

### Data collection

**Table:**

Enraf–Nonius CAD-4 diffractometer	1422 reflections with *I* > 2σ(*I*)
Radiation source: fine-focus sealed tube	*R*_int_ = 0.048
graphite	θ_max_ = 25.2°, θ_min_ = 1.8°
ω/2θ scans	*h* = −22→20
Absorption correction: ψ scan (North *et al.*, 1968)	*k* = 0→18
*T*_min_ = 0.973, *T*_max_ = 0.982	*l* = 0→10
2123 measured reflections	3 standard reflections every 200 reflections
2058 independent reflections	intensity decay: none

### Refinement

**Table:**

Refinement on *F*^2^	Primary atom site location: structure-invariant direct methods
Least-squares matrix: full	Secondary atom site location: difference Fourier map
*R*[*F*^2^ > 2σ(*F*^2^)] = 0.055	Hydrogen site location: difference Fourier map
*wR*(*F*^2^) = 0.164	H-atom parameters constrained
*S* = 1.00	*w* = 1/[σ^2^(*F*_o_^2^) + (0.080*P*)^2^ + 2.*P*] where *P* = (*F*_o_^2^ + 2*F*_c_^2^)/3
2058 reflections	(Δ/σ)_max_ < 0.001
146 parameters	Δρ_max_ = 0.21 e Å^−^^3^
0 restraints	Δρ_min_ = −0.27 e Å^−^^3^

### Special details

**Table:**

Geometry. All e.s.d.'s (except the e.s.d. in the dihedral angle between two l.s. planes) are estimated using the full covariance matrix. The cell e.s.d.'s are taken into account individually in the estimation of e.s.d.'s in distances, angles and torsion angles; correlations between e.s.d.'s in cell parameters are only used when they are defined by crystal symmetry. An approximate (isotropic) treatment of cell e.s.d.'s is used for estimating e.s.d.'s involving l.s. planes.
Refinement. Refinement of *F*^2^ against ALL reflections. The weighted *R*-factor *wR* and goodness of fit *S* are based on *F*^2^, conventional *R*-factors *R* are based on *F*, with *F* set to zero for negative *F*^2^. The threshold expression of *F*^2^ > σ(*F*^2^) is used only for calculating *R*-factors(gt) *etc*. and is not relevant to the choice of reflections for refinement. *R*-factors based on *F*^2^ are statistically about twice as large as those based on *F*, and *R*- factors based on ALL data will be even larger.

### Fractional atomic coordinates and isotropic or equivalent isotropic displacement parameters (Å^2^)

**Table:**

	*x*	*y*	*z*	*U*_iso_*/*U*_eq_	
O1	0.5000	0.43398 (16)	0.2500	0.0707 (8)	
C1	0.5000	0.3537 (2)	0.2500	0.0510 (8)	
C2	0.47174 (14)	0.29645 (15)	0.3510 (3)	0.0480 (6)	
O2	0.36310 (14)	0.07554 (11)	0.6941 (3)	0.0806 (7)	
O3	0.32676 (10)	0.18198 (11)	0.89316 (19)	0.0592 (5)	
C3	0.47864 (15)	0.20225 (14)	0.3086 (3)	0.0512 (6)	
H3A	0.4268	0.1760	0.2546	0.061*	
H3B	0.5085	0.1690	0.4080	0.061*	
O4	0.33734 (12)	0.35568 (11)	0.8758 (2)	0.0683 (6)	
C4	0.44589 (14)	0.33133 (16)	0.4587 (3)	0.0517 (6)	
H4A	0.4489	0.3922	0.4665	0.062*	
C5	0.41371 (14)	0.28904 (15)	0.5662 (3)	0.0473 (6)	
C6	0.39179 (15)	0.34335 (15)	0.6677 (3)	0.0526 (6)	
H6A	0.3979	0.4038	0.6648	0.063*	
C7	0.36086 (14)	0.30734 (16)	0.7728 (3)	0.0504 (6)	
C8	0.35313 (13)	0.21799 (15)	0.7816 (3)	0.0478 (6)	
C9	0.37410 (15)	0.16327 (15)	0.6797 (3)	0.0542 (6)	
C10	0.40400 (15)	0.19887 (16)	0.5729 (3)	0.0555 (6)	
H10A	0.4177	0.1620	0.5050	0.067*	
C11	0.3772 (3)	0.01797 (19)	0.5835 (5)	0.1075 (13)	
H11A	0.3679	−0.0414	0.6071	0.161*	
H11B	0.4309	0.0240	0.5965	0.161*	
H11C	0.3425	0.0323	0.4718	0.161*	
C12	0.24333 (17)	0.1766 (2)	0.8306 (4)	0.0739 (8)	
H12A	0.2284	0.1507	0.9126	0.111*	
H12B	0.2239	0.1410	0.7326	0.111*	
H12C	0.2212	0.2345	0.8044	0.111*	
C13	0.3430 (2)	0.44758 (18)	0.8706 (4)	0.0886 (11)	
H13A	0.3249	0.4735	0.9478	0.133*	
H13B	0.3111	0.4681	0.7611	0.133*	
H13C	0.3969	0.4639	0.8995	0.133*	

### Atomic displacement parameters (Å^2^)

**Table:**

	*U*^11^	*U*^22^	*U*^33^	*U*^12^	*U*^13^	*U*^23^
O1	0.118 (2)	0.0480 (15)	0.0791 (18)	0.000	0.0745 (17)	0.000
C1	0.074 (2)	0.048 (2)	0.0498 (18)	0.000	0.0438 (17)	0.000
C2	0.0656 (14)	0.0492 (13)	0.0459 (12)	−0.0033 (10)	0.0397 (11)	−0.0017 (10)
O2	0.1371 (18)	0.0460 (11)	0.0952 (15)	−0.0030 (10)	0.0846 (14)	0.0043 (9)
O3	0.0752 (12)	0.0681 (11)	0.0527 (10)	−0.0055 (9)	0.0450 (9)	0.0105 (8)
C3	0.0719 (16)	0.0494 (13)	0.0494 (13)	−0.0013 (11)	0.0422 (12)	0.0001 (10)
O4	0.1063 (14)	0.0569 (11)	0.0782 (12)	−0.0088 (9)	0.0749 (12)	−0.0123 (9)
C4	0.0734 (16)	0.0497 (14)	0.0515 (13)	0.0006 (11)	0.0452 (12)	0.0011 (10)
C5	0.0631 (14)	0.0482 (13)	0.0463 (12)	−0.0029 (10)	0.0384 (11)	−0.0005 (10)
C6	0.0770 (16)	0.0447 (13)	0.0575 (14)	−0.0010 (11)	0.0493 (13)	0.0004 (10)
C7	0.0666 (15)	0.0550 (14)	0.0474 (12)	−0.0045 (11)	0.0414 (12)	−0.0058 (10)
C8	0.0585 (14)	0.0542 (14)	0.0433 (12)	−0.0037 (11)	0.0336 (11)	0.0049 (10)
C9	0.0779 (17)	0.0449 (13)	0.0564 (14)	−0.0038 (11)	0.0443 (13)	0.0040 (11)
C10	0.0807 (17)	0.0506 (14)	0.0556 (14)	0.0013 (12)	0.0487 (13)	−0.0018 (11)
C11	0.188 (4)	0.0482 (18)	0.130 (3)	−0.005 (2)	0.109 (3)	−0.0127 (18)
C12	0.0775 (19)	0.085 (2)	0.0795 (19)	−0.0150 (15)	0.0526 (16)	0.0035 (16)
C13	0.149 (3)	0.0538 (17)	0.108 (2)	−0.0021 (18)	0.098 (2)	−0.0120 (16)

### Geometric parameters (Å, °)

**Table:**

O1—C1	1.222 (4)	C5—C6	1.398 (3)
C1—C2	1.489 (3)	C6—C7	1.389 (3)
C1—C2^i^	1.489 (3)	C6—H6A	0.9300
C2—C4	1.339 (3)	C7—C8	1.374 (3)
C2—C3	1.501 (3)	C8—C9	1.395 (3)
O2—C9	1.365 (3)	C9—C10	1.386 (3)
O2—C11	1.415 (3)	C10—H10A	0.9300
O3—C8	1.381 (2)	C11—H11A	0.9600
O3—C12	1.420 (3)	C11—H11B	0.9600
C3—C3^i^	1.541 (4)	C11—H11C	0.9600
C3—H3A	0.9700	C12—H12A	0.9600
C3—H3B	0.9700	C12—H12B	0.9600
O4—C7	1.373 (3)	C12—H12C	0.9600
O4—C13	1.406 (3)	C13—H13A	0.9600
C4—C5	1.462 (3)	C13—H13B	0.9600
C4—H4A	0.9300	C13—H13C	0.9600
C5—C10	1.390 (3)		
			
O1—C1—C2	125.87 (13)	C7—C8—O3	120.62 (19)
O1—C1—C2^i^	125.87 (13)	C7—C8—C9	119.49 (19)
C2—C1—C2^i^	108.3 (3)	O3—C8—C9	119.9 (2)
C4—C2—C1	120.7 (2)	O2—C9—C10	124.2 (2)
C4—C2—C3	130.4 (2)	O2—C9—C8	115.7 (2)
C1—C2—C3	108.86 (18)	C10—C9—C8	120.1 (2)
C9—O2—C11	117.7 (2)	C9—C10—C5	120.7 (2)
C8—O3—C12	113.10 (19)	C9—C10—H10A	119.7
C2—C3—C3^i^	106.72 (11)	C5—C10—H10A	119.7
C2—C3—H3A	110.4	O2—C11—H11A	109.5
C3^i^—C3—H3A	110.4	O2—C11—H11B	109.5
C2—C3—H3B	110.4	H11A—C11—H11B	109.5
C3^i^—C3—H3B	110.4	O2—C11—H11C	109.5
H3A—C3—H3B	108.6	H11A—C11—H11C	109.5
C7—O4—C13	117.66 (19)	H11B—C11—H11C	109.5
C2—C4—C5	130.4 (2)	O3—C12—H12A	109.5
C2—C4—H4A	114.8	O3—C12—H12B	109.5
C5—C4—H4A	114.8	H12A—C12—H12B	109.5
C10—C5—C6	118.77 (19)	O3—C12—H12C	109.5
C10—C5—C4	123.87 (19)	H12A—C12—H12C	109.5
C6—C5—C4	117.4 (2)	H12B—C12—H12C	109.5
C7—C6—C5	120.3 (2)	O4—C13—H13A	109.5
C7—C6—H6A	119.9	O4—C13—H13B	109.5
C5—C6—H6A	119.9	H13A—C13—H13B	109.5
O4—C7—C8	115.13 (18)	O4—C13—H13C	109.5
O4—C7—C6	124.2 (2)	H13A—C13—H13C	109.5
C8—C7—C6	120.7 (2)	H13B—C13—H13C	109.5
			
O1—C1—C2—C4	−2.7 (3)	O4—C7—C8—O3	−2.8 (3)
C2^i^—C1—C2—C4	177.3 (3)	C6—C7—C8—O3	176.1 (2)
O1—C1—C2—C3	177.49 (12)	O4—C7—C8—C9	178.8 (2)
C2^i^—C1—C2—C3	−2.51 (12)	C6—C7—C8—C9	−2.2 (4)
C4—C2—C3—C3^i^	−173.4 (3)	C12—O3—C8—C7	86.9 (3)
C1—C2—C3—C3^i^	6.4 (3)	C12—O3—C8—C9	−94.7 (3)
C1—C2—C4—C5	178.0 (2)	C11—O2—C9—C10	−5.3 (4)
C3—C2—C4—C5	−2.2 (5)	C11—O2—C9—C8	174.8 (3)
C2—C4—C5—C10	−0.3 (4)	C7—C8—C9—O2	−178.8 (2)
C2—C4—C5—C6	179.6 (3)	O3—C8—C9—O2	2.9 (4)
C10—C5—C6—C7	0.0 (4)	C7—C8—C9—C10	1.3 (4)
C4—C5—C6—C7	−180.0 (2)	O3—C8—C9—C10	−177.0 (2)
C13—O4—C7—C8	−179.2 (3)	O2—C9—C10—C5	−179.7 (2)
C13—O4—C7—C6	1.8 (4)	C8—C9—C10—C5	0.2 (4)
C5—C6—C7—O4	−179.6 (2)	C6—C5—C10—C9	−0.8 (4)
C5—C6—C7—C8	1.6 (4)	C4—C5—C10—C9	179.1 (2)

Symmetry codes: (i) −*x*+1, *y*, −*z*+1/2.
